# Correspondence

**Published:** 1912-08

**Authors:** W. A. MacPherson

**Affiliations:** Tonawanda, N. Y.


Mr. Editor:—

Seeing various cases reported in medical journals (*N. Y. Med. Jour.*, Mar., 1912; *Bfo. Med. Journ.*, Apr., 1912) regarding results obtained from use of Dowd's Phosphatometer and Phosphorus Tonic, stimulates me to offer you a case reported for publication that to my mind is as important as, if not more so than those already reported.

Mrs. S., aged 29, married.

A more typical case of articular rheumatism it would be hard to find than presented by Mrs. S. when I called to see her. She had been ailing for some time and upon my first visit presented swollen joints with severe pain most typical of rheumatism. She was unable to raise her arms above the waist line and her feet were so tender and swollen that it was necessary for her to wear shoes two sizes larger than normal.

Under the most careful treatment and diet she did not improve and I advised her to go to a mineral spring quite noted as a cure for rheumatism. She remained there for six weeks but instead of improving she grew worse and finally returned home.

Having heard the remark of Dr. J. Henry Dowd of Buffalo that many cases of pain supposed to be due to rheumatism, were not rheumatic pains at all, but the nerves crying for food, I sent her to Buffalo for consultation.

Dr. Dowd reported to me that after careful examination he found albumin due to anemia; no pathological condition, but an alkaline urine with a phosphatic index scarcely perceptible, about 96% minus; that the condition was neurainidia, nerve cell starvation, with a marked hysterical element.

She was put on the Dowd Phosphorus Tonic with valerian and improvement was evident in a few days. In four or five weeks she was entirely free from pain, could dress herself, in fact was practically well and had gained 95 pounds. (Four months after, she was well, no pain and in first class condition.)

In several other cases I have had most gratifying results from this tonic, especially where I have used it in connection with the Phosphatometer.

Yours truly,
W. A. MacPHERSON, M. D.

Tonawanda, N. Y.

